# Phase transformation and subsurface damage formation in the ultrafine machining process of a diamond substrate through atomistic simulation

**DOI:** 10.1038/s41598-021-97419-9

**Published:** 2021-09-07

**Authors:** Van-Thuc Nguyen, Te-Hua Fang

**Affiliations:** 1 Department of Mechanical Engineering, National Kaohsiung University of Science and Technology, Kaohsiung, 807 Taiwan; 2 Faculty of Mechanical Engineering, Ho Chi Minh City University of Technology and Education, Ho Chi Minh City, Vietnam

**Keywords:** Materials science, Nanoscience and technology

## Abstract

This report explores the effects of machining depth, velocity, temperature, multi-machining, and grain size on the tribological properties of a diamond substrate. The results show that the appearance of graphite atoms can assist the machining process as it reduces the force. Moreover, the number of graphite atoms relies on the machining speed and substrate temperature improvement caused by the friction force. Besides, machining in a machined surface for multi-time is affected by its rough, amorphous, and deformed surface. Therefore, machining in the vertical direction for multi-time leads to a higher rate of deformation but a reduction in the rate of graphite atoms generation. Increasing the grain size could produce a larger graphite cluster, a higher elastic recovery rate, and a higher temperature but a lower force and pile-up height. Because the existence of the grain boundaries hinders the force transformation process, and the reduction in the grain size can soften the diamond substrate material.

## Introduction

Diamond thin film is considered one of the most attractive materials for coating due to its advantage of high Young’s modulus, extreme hardness, high wear resistance, low friction coefficient, and outstanding chemical stability^1–3^. Moreover, this type of film is a promising candidate for semiconductor applications because of its wide bandgap and high thermal conductivity^4–6^. Besides single crystal film, the polycrystalline diamond film also receives vigorous attention because of its excellent hardness compared to the single crystal one^7^.

A diamond film must undergo conventional processes such as generating, grinding, and polishing before being used in further applications^8^. Thus, despite the advantageous characteristics, diamond film application is still limited due to the challenges in machining an extremely hard material like this. Many studies focus on researching the characteristics of a diamond film to improve machining effectiveness. For instance, Zong et al.^9^ indicated the material removal rate of different orientations at the atomic level by employing molecular dynamics (MD) simulation. While He et al.^10^ investigated the indentation process and pointed out the temperature effect on the mechanical response of the nanocrystalline diamond material. Thomas et al.^11^ argued that the surface roughness of the film could be reduced up to 1.7 nm by using chemical mechanical polishing. By using a sol–gel pad combines with diamond abrasive particles in the chemical mechanical polishing process, the diamond film surface roughness could reach 1.32 nm^12^. Yang et al.^13^ reported that the stick–slip phenomenon could be found in the deformed surface layer, showing the lubricant behavior of the diamond surface in a sliding motion. Remarkably, Roy et al.^14^ proved that using a suitable combination of abrasive types and mechanical polishing can obtain a good surface roughness for sensor or photonic devices. With the presence of an upper amorphous layer, the maximum stress zone appears near the cutting surface in a banded shape during the lapping process^15^.

The existence of an amorphous layer in the machining process of a diamond film is inevitable. This layer is continuously created during the material removal process. The forming of the amorphous layer negatively influences the quality of the diamond film, reducing the electro-optical characteristics of the material. However, this topic has not thoroughly examined yet. This report will pay attention to the phase transformation, graphitization, and subsurface damage (SSD) in the scratching process of a single crystal diamond substrate by using the MD simulation method. We also survey a polycrystalline diamond substrate to explore its tribological properties. We also conduct a multi-scratches study to imitate the continuous machining process. Section “Effect of depth and temperature” presents a single crystal diamond substrate investigation in different machining velocities, depths, and temperatures. While “Effect of crystalline orientation” examines the effect of crystalline orientation. Sections “Multi-machining” and “Polycrystalline” survey the multi-scratches model and polycrystalline model, respectively. Section “Discussion” shows the computational method for the report.

## Results

### Effect of depth and temperature

**Figure 1:**
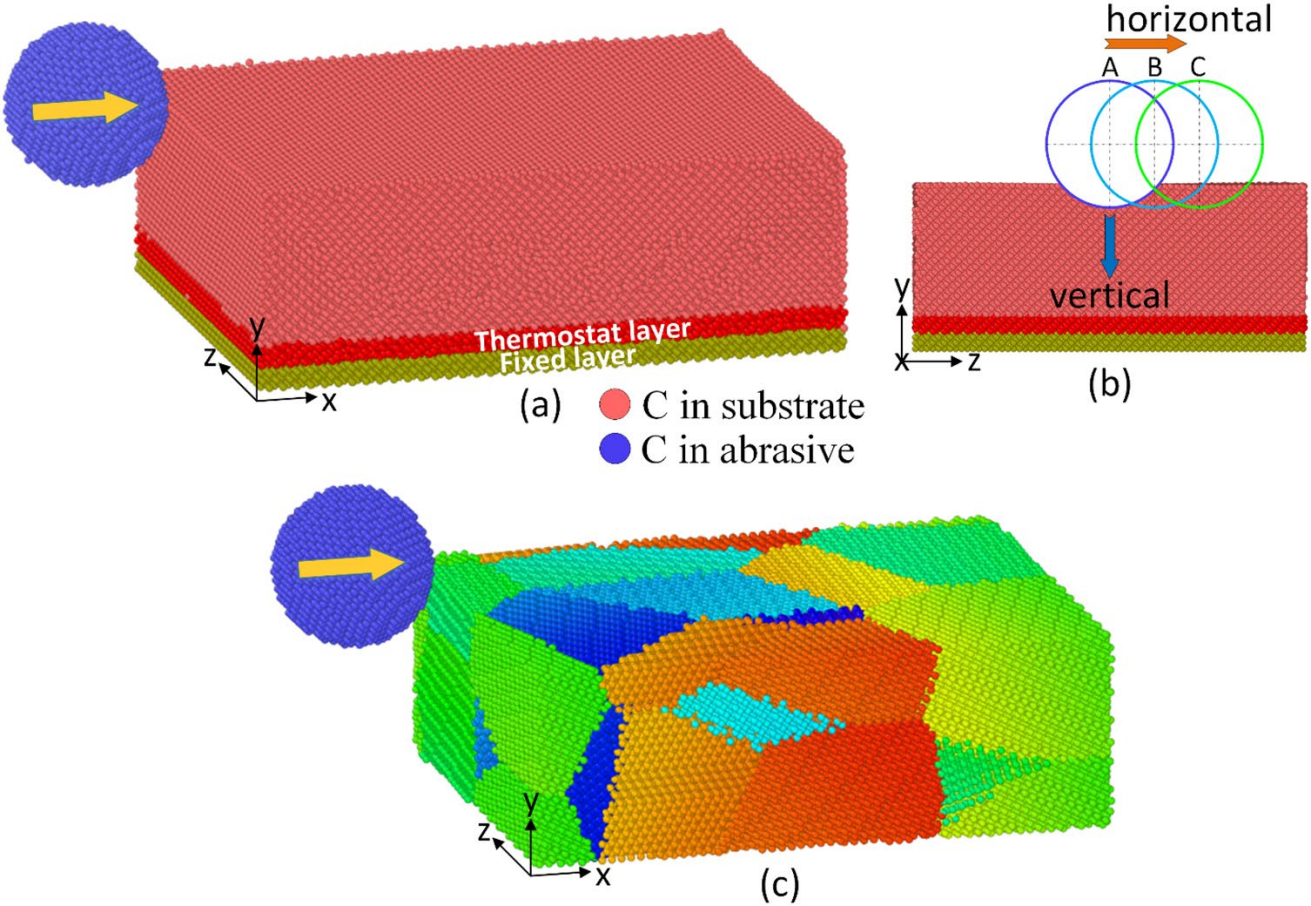
The simulation models: (**a**) single crystal diamond, (**b**) multi-machining, and (**c**) polycrystalline diamond.

This section surveys the effect of machining depths and temperatures on the machining process of a single crystal diamond substrate, as aforementioned. The simulation model is shown in Fig. 1a–c. The crystalline orientation of the diamond workpiece that the abrasive machines is selected as (100)[100]. When considering the effect of machining depth, the temperature and velocity are fixed at 300 K and 100 m/s. Then, the effect of substrate temperature is examined by setting at 100 m/s and 10 Å depth.

**Figure 2:**
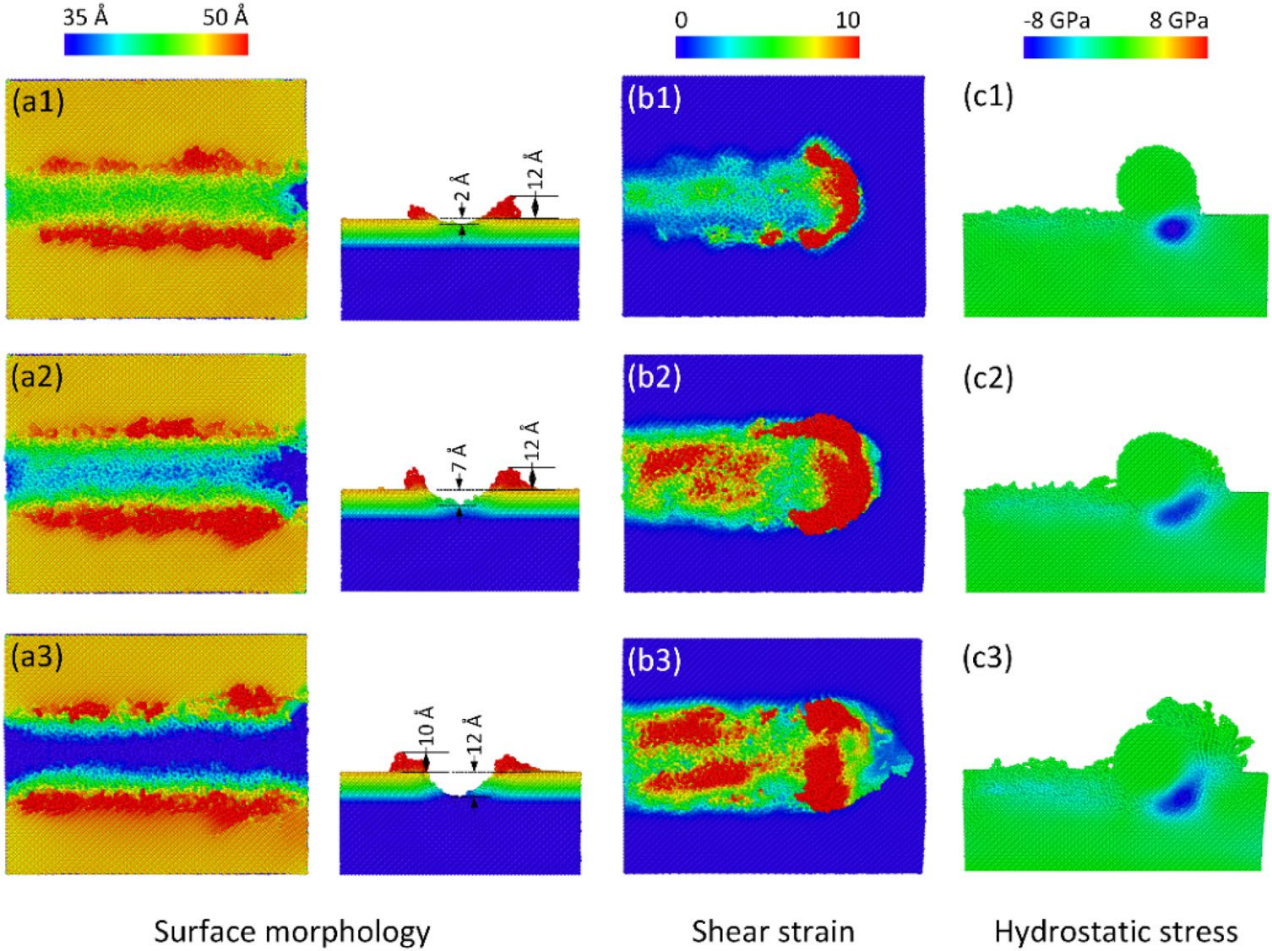
Surface morphology, shear strain and hydrostatic stress distributions of different depths at 300 K, 100 m/s: (**a1**, **c1**) 5 Å, (**a2**, **c2**) 10 Å, and (**a3**, **c3**) 15 Å.

Firstly, the effect of depths on the machining process of the diamond substrate is investigated. Figure 2 shows the surface morphology, shear strain, and hydrostatic stress distributions of the workpiece of different depths at 300 K, 100 m/s. The results exhibit that the deeper the tooltip intrudes the substrate, the deeper the groove depth, as shown in Fig. 2a1–a3. The reason is the stronger impact of the abrasive on the workpiece when increasing the depth, causing a greater deformation level. While the protrusion height oscillates around 10–12 Å when surging the depth, expressing a weak dependence between it and the depth. In addition, increasing the depth leads to a surge in the shear strain, hydrostatic stress, and protrusion height due to the more significant deformation level. At 5 Å depth, the high-strain zone mainly concentrates on the front side of the tooltip. From 10 Å depth, besides that position, the substrate atoms are also strongly displaced on the pathway, as exhibited in Fig. 2b2,b3. Notably, when the tooltip intrudes deeper into the substrate, the high-stress zone would become broader and tend to rise to the surface because of a greater deformation rate, as exhibited in Fig. 2c1–c3. This high-stress status can lead to a graphitization process of the diamond workpiece because diamond can experience a phase transformation for which graphitic carbons is created under pressure^16^.

**Figure 3:**
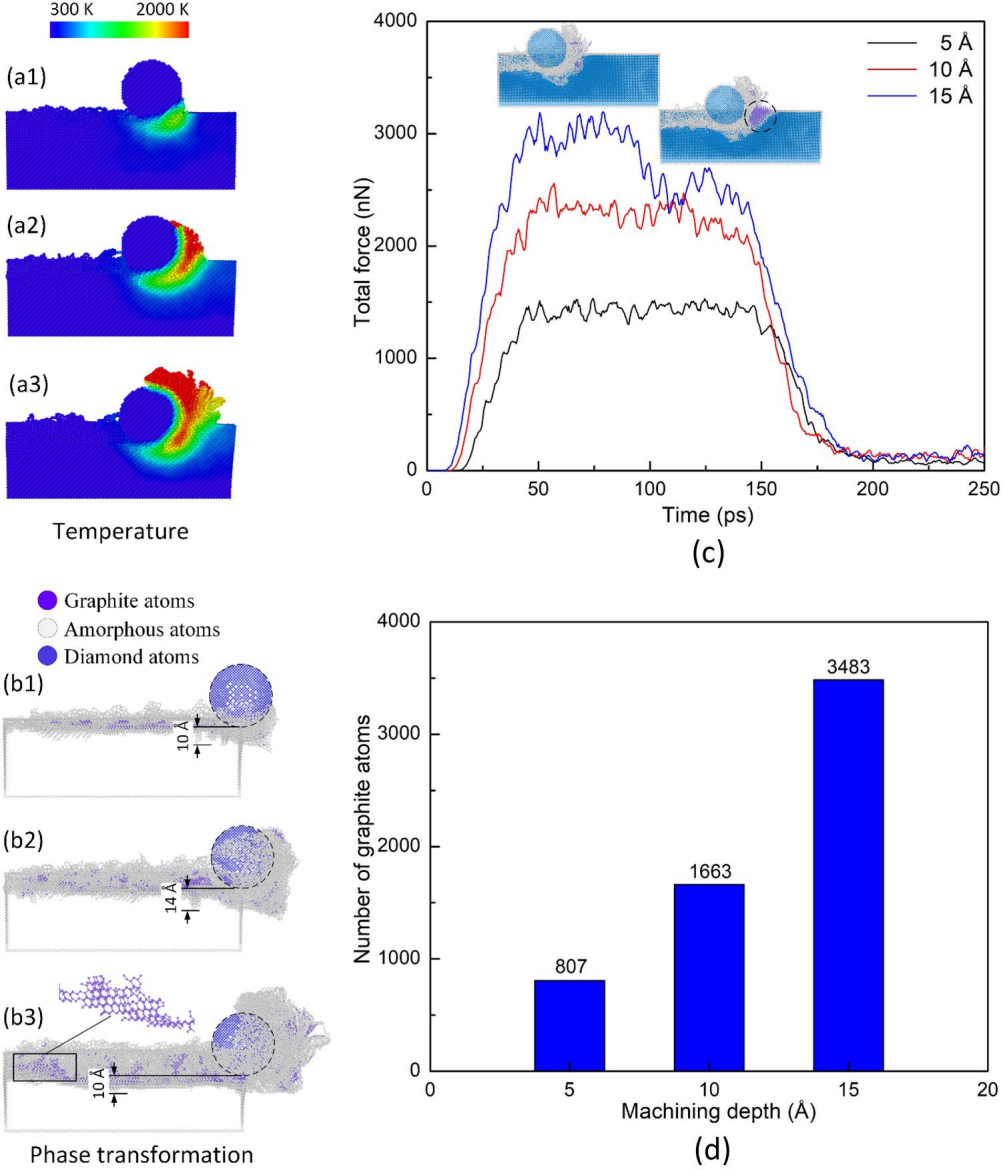
Temperature distribution, phase transformation, force diagram, and number of graphite atoms of different depths at 300 K, 100 m/s: (**a1**, **b1**) 5 Å, (**a2**, **b2**) 10 Å, and (**a3**, **b3**) 15 Å; (**c**) total force; (**d**) number of graphite atoms vs depth.

Figure 3 shows the temperature distribution, phase transformation, force diagram, and the graphite atoms number of the diamond workpiece of different machining depths at 300 K, 100 m/s. Overall, increasing the depth leads to a rapid rise in the substrate temperature, especially at the front side of the tooltip, due to the increase in the impact force between the substrate and the tooltip. Figure 3c provides evidence for the rise of the force when increasing the depth, thus, causing a higher deformation rate and substrate temperature. Under high pressure, the diamond substrate can be transformed into an amorphous state or a graphene structure^1^. Besides the role of high pressure, the high-temperature state generated by the tooltip movement also contributes to the graphitization process^2^, creating sheets of graphite atoms along the pathway, as shown in Fig. 3b3. Figure 3b1–b3 show that the highest SSD value of the diamond workpiece varies around 10–14 Å. Although the deeper machining depth does not result in a higher highest SSD value, the average value of the SSD depths increases when the tooltip penetrates deeper into the substrate due to the stronger impact of the tooltip. Notably, improving the machining depth results in a higher force due to a higher collision rate between the tooltip and the diamond substrate, as shown in Fig. 3c. This is the explanation for the improvement of the groove depth, deformation rate, strain, stress, and graphitization process. As aforementioned, during the machining process of the diamond substrate, the diamond structure can be transformed into a graphite structure via the graphitization process. The diagram in Fig. 3d shows that the numbers of graphite atoms are 807, 1663, and 3483 atoms corresponding to 5 Å, 10 Å, and 15 Å depths. The number of graphene atoms rapidly rises when increasing the machining depth due to the surge in the applied stress. At 15 Å depth, the sharp rise in the number of graphite atoms during machining causes a sudden drop in the total force, as shown in Fig. 3c. In this case, the number of graphite atoms is sufficient to form sizeable graphite sheets rather than small of clusters graphite atoms, as shown in Fig. 3b1–b3. Because the Van der Waals interactions between the graphite sheets are weak, they are easily separated under a low force, facilitating the removal process of a diamond substrate^17^.

Besides the machining depth, this section also inspects the effect of the substrate temperature. Supplementary Fig. 1 shows the shear strain and shear stress distributions of different substrate temperatures at 100 m/s and 10 Å depth. Similar to the effect of machining speed, the shear strain does not strongly depend on the diamond substrate temperature, as shown in Supplementary Fig. 1a1–d1. Meanwhile, the high-stress zones seem to turn into slimmer shapes when heightening the substrate temperature, as shown in Supplementary Fig. 1a2–d2. In other words, these zones mainly obtain a shallower depth when raising the temperature. Because the higher substrate temperature softens the diamond substrate due to the weakening of the interatomic bonding^18,19^, the substrate can release the stress more effectively at a higher temperature than the lower one has more thermal vibration.

**Figure 4:**
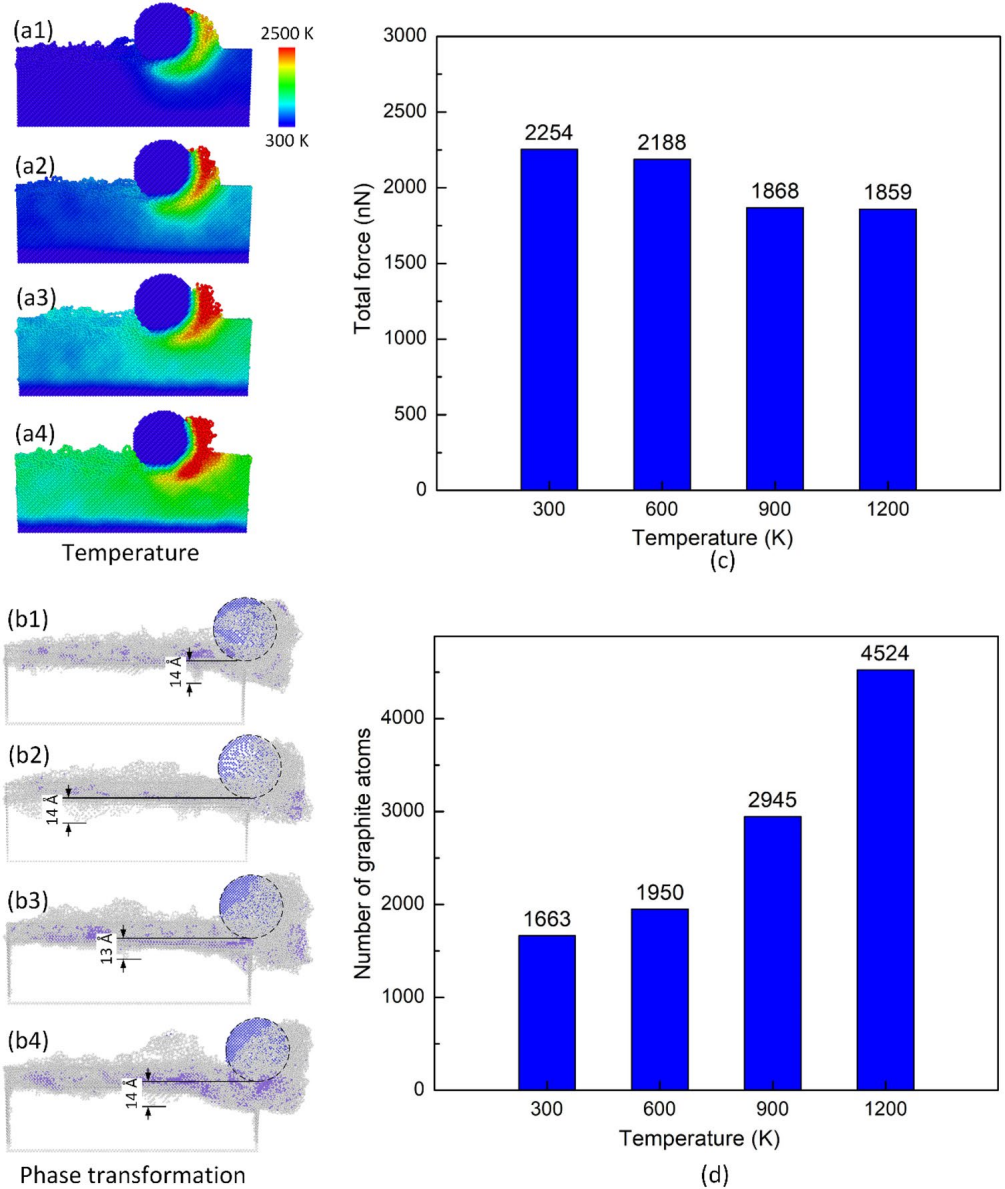
Temperature distribution, phase transformation, force diagram, and number of graphite atoms of different temperatures at 100 m/s, 10 Å depth: (**a1**, **b1**) 300 K, (**a2**, **b2**) 600 K, (**a3**, **b3**) 900 K, and (**a4**, **b4**) 1200 K; (**c**) total force vs temperature; (**d**) number of graphite atoms vs temperature.

Figure 4 displays the temperature distribution, phase transformation of different substrate temperatures at 100 m/s, 10 Å depth. Generally, raising the substrate temperature induces a higher temperature of the deformed zone than the around area. Because with the higher original temperature, the substrate has more advantage to reach a higher temperature when machining^20^. Increasing the temperature does not lead to an appreciable change in the pile-up height. The SSD values fluctuate around 13–14 Å, therefore, improving the temperature also does not cause much change in SSD depths. Figure 4c,d show the total force and the number of graphite atoms of different substrate temperatures at 100 m/s, 10 Å depth. The total force values are 2254 nN, 2188 nN, 1868 nN, and 1859 nN, correlating to 300 K, 600 K, 900 K, and 1200 K. The force slightly reduces when raising the substrate temperature because the substrate becomes softer at a higher temperature^21–23^. At a higher temperature, the atoms vibrate at a faster rate, the bonding strength between them is weakened, leading to a softer material. The numbers of graphite atoms are 1663, 1950, 2945, and 4524 atoms, corresponding to 300 K, 600 K, 900 K, and 1200 K. The number of graphite atoms grows when the substrate temperature escalates. From 300 to 600 K, the number of graphite atoms grows moderately. Otherwise, from 600 to 1200 K, the number of graphite atoms dramatically surge when escalating the substrate temperature because the rapid improvement of the substrate temperature facilitates the graphitization process. The diamond structure is a metastable allotrope of carbon, it can be converted to a more stable state like graphite through the graphitization process by suffering high temperature and high pressure. Additionally, the deformed zone temperature in front of the tooltip dramatically rises when heightening the substrate temperature, as shown in Fig. 4a2–a4.

### Effect of crystalline orientation

This section examines the anisotropy of the diamond workpiece in the machining process by surveying this process in different orientations of (100)[100], (100)[110], (110)[100], (110)[110]. The tooltip moves at a speed of 100 m/s, a 10 Å depth, while the workpiece temperature is 300 K.

**Figure 5:**
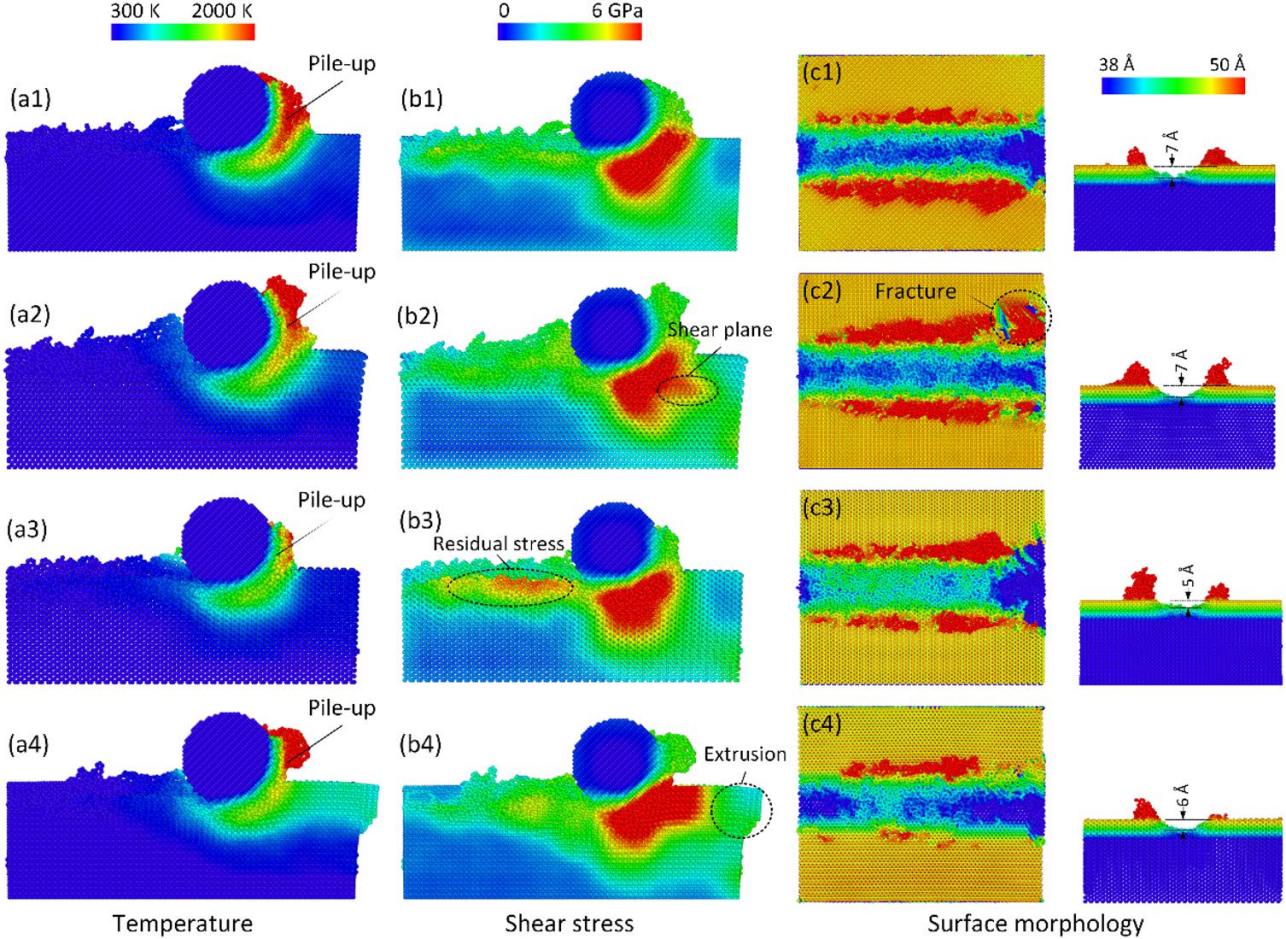
Temperature, shear stress distributions, and surface morphology of different orientations at 300 K, 100 m/s, and 10 Å depth: (**a1**–**c1**) (100)[100], (**a2**–**c2**) (100)[110], (**a3**–**c3**) (110)[100], and (**a4**–**c4**) (110)[110].

Figure 5 exhibits the diamond workpiece's temperature and shear stress distributions of different orientations at 300 K, 100 m/s, and 10 Å depth. Figure 5a3 displays that orientation (110)[100] reaches a considerably lower temperature than the other orientations. At the same time, the pile-up height in this orientation is the lowest among all orientations. Moreover, the groove depth of this orientation is the shallowest value, as shown in Fig. 5c3. These reasons indicate that orientation (110)[100] has the strongest rate of elastic recovery. In other words, this orientation has the lowest level of plastic deformation. This is why the residual stress appearing along the pathway of this orientation is higher than the other orientations, as shown in Fig. 5b3. On the contrary, orientation (100)[110] achieves the highest pile-up and the deepest groove, as presented in Fig. 5a2,c2, pointing out the highest level of plastic deformation. Besides, the shear stress distribution of this orientation shows the split of the high-stress zone and the shear plane, indicated the scattering of the stress distribution, as presented in Fig. 5b2. That could be the reason why this orientation appears a fracture at the right edge of the workpiece, pointing out severe damage on the surface, as shown in Fig. 5c2. Similar to orientation (100)[110], orientation (100)[100] has the deepest groove value of 7 Å and a high rate of protrusion along the pathway, as shown in Fig. 5c1,c2. This orientation also obtains a high pile-up. Nevertheless, the shear stress distribution of this orientation is not separated like orientation (100)[110]. Therefore, its surface does not appear to fracture like orientation (100)[110]. In orientation (110)[110], the high-stress zone is separated into two parts, as shown in Fig. 5b4. The first part locates right below the abrasive, while the second one distributes along the workpiece surface. The high pressure along the surface generates an extrusion part or shear band at the edge of the workpiece to release the stress. The groove depth values of orientations (100)[100], (100)[110], (110)[100], and (110)[110] are 7, 7, 5, and 6 Å depth, respectively, as shown in Fig. 5c1–c4. These results indicate that orientations (110)[100] and (110)[110] have better recovery rates than the orientations (100)[100] and (100)[110]. The protrusion rates along the pathway also identify that conclude.

**Figure 6:**
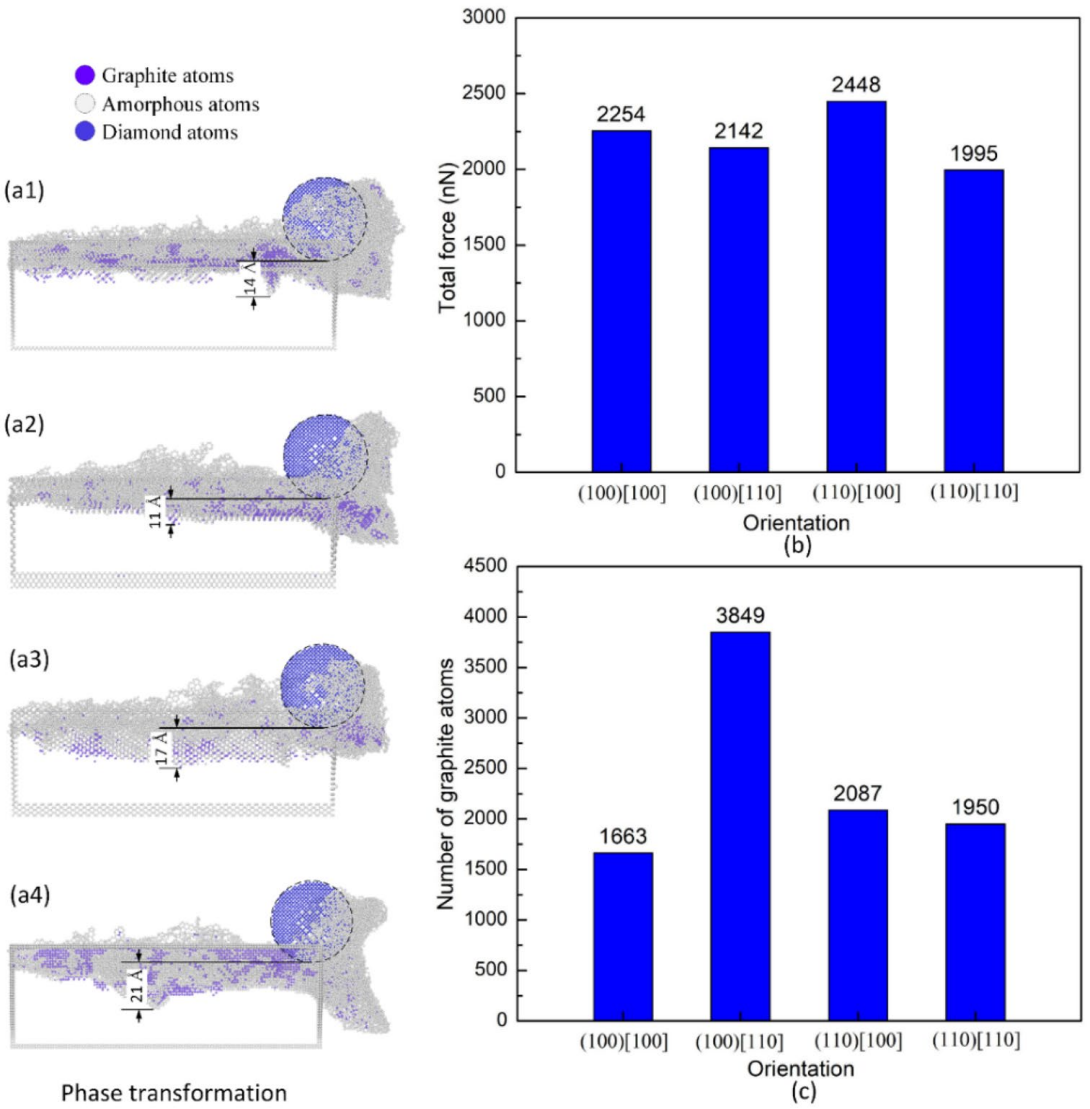
Phase transformation, total force and number of graphite atoms of different orientations at 300 K, 100 m/s, and 10 Å depth: (**a1**) (100)[100], (**a2**) (100)[110], (**a3**) (110)[100], and (**a4**) (110)[110]; (**b**) total force vs orientation; (**c**) number of graphite atoms vs orientation.

Figure 6a1–a4 show the phase transformation of the diamond workpiece of different orientations at 300 K, 100 m/s, and 10 Å depth. The SSD depths of orientations (100)[100], (100)[110], (110)[100], and (110)[110] are 14 Å, 11 Å, 17 Å, and 21 Å, respectively. Orientation (110)[110] generated the deepest SSD due to the strong deformation of the workpiece with high pile-up and extrusion, as shown in Fig. 5b4. Orientation (100)[110] produces the shallowest SSD as a result of the division of the high-stress zone by the shear plane, as exhibited in Fig. 5b2. Besides, the orientation (110)[100] has a deeper SSD than (100)[100] due to the high-stress zone concentrate deeper below the tooltip, as presented in Fig. 5b3. The volume of protrusion along the pathway in Fig. 5c1–c4 pinpoints that the lesser the protrusion volume, the deeper the SSD depth. Because when the material is harder to remove from the groove to form protrusion or more challenging to deform plastically, it tends to be compressed more. Figure 6b,c display the total force and the number of graphite atoms of different orientations at 300 K, 100 m/s, and 10 Å depth. The total force values are 2254 nN, 2142 nN, 2448 nN, and 1995 nN corresponding to orientations (100)[100], (100)[110], (110)[100], and (110)[110], respectively. Orientation (110)[100] requires the highest force value due to the strong elastic ability of this orientation, as shown in Fig. 5c3. While the lowest force exposes in orientation (110)[110] due to the appearance of the extrusion part, as shown in Fig. 5b4. This deformation part help release the stress and reduce the required force. However, the differences between those forces are not so huge. The number of graphite atoms generated are 1663, 3849, 2087, and 1950 atoms corresponding to orientations (100)[100], (100)[110], (110)[100], and (110)[110], respectively. Remarkably, the orientation (100)[110] obtains the highest number of graphite atoms, which is significantly higher than the other orientations. The reason is the natural anisotropy characteristic of a crystalline structure as a different orientation has its own arrangement order. This transformation releases the energy of the sliding process, leading to the lowest SSD value of this orientation.

### Multi-machining

In this section, we consider the machining process by sliding the abrasive on a single crystal diamond substrate many times and in many positions, as shown in Fig. 1b. In the lapping and polishing processes, the abrasive particles are continuously added and removed. Therefore, after machining, the new abrasive will replace the old one. We survey the multi-machining process in the vertical direction. In the beginning, the abrasive intrudes at 5 Å depth. After that, it moves down to operate at 10 Å depth in the vertical direction. Then, the abrasive moves down again to 15 Å depth. Then, a new abrasive for the second machining time is created. Finally, we also repeat the previous steps by removing the abrasive with its bonded diamond atoms and create a new abrasive to machining in the two-time machined workpiece for the third time.

**Figure 7:**
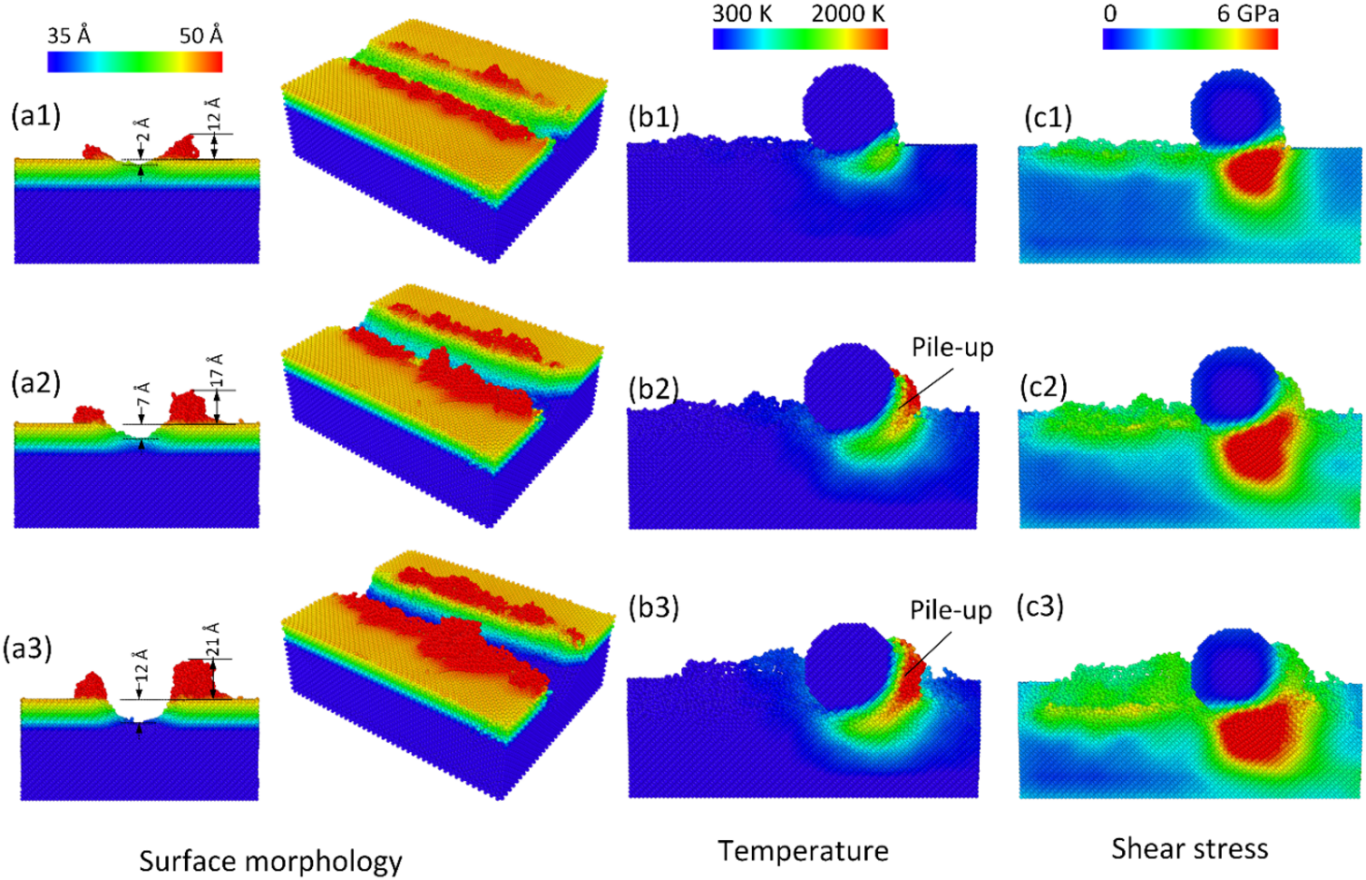
Surface morphology, temperature, and shear stress distributions when applying multi-machining in the vertical direction at 300 K and 100 m/s: (**a1**–**c1**) first time, (**a2**–**c2**) second time, and (**a3**–**c3**) third time.

Figure 7a1–a3 display the surface morphology when applying three-time machining in the vertical direction at 300 K and 100 m/s. We ignore the edge effect by removing the extension part at the end of the substrate. The results indicate that the protrusions along the pathway are heightened quickly due to the accumulation of the previous machining times. Hence, the protrusion heights of the second and the third machining times are greatly higher than the single machining cases, as shown in Fig. 2a1–a3. Besides, after machining at the same position for multi-time, a deeper groove is generated on the substrate surface. The groove depth values are 2 Å, 7 Å, 12 Å, corresponding to the first time, the second time, and the third time of machining. Interestingly, the groove depths of the multi-machining cases and the single machining cases with different depths have equivalent values, as shown in Fig. 2a1–a3. This phenomenon indicates the same elastic deformation rate. In other words, the elastic deformation rate does not depend on the single or multi-machining cases. Figure 7b1–c3 show the temperature and shear stress distributions when applying multi-machining in the vertical direction at 300 K, 100 m/s. At the same 5 Å intrusion depth for each machining time, increasing the machining time results in a higher rate of temperature and pile-up. Notably, it is different from increasing the machining depth leading to the higher temperature, as mentioned in “Effect of depth and temperature”. This increase causes by more substrate atoms cover around the abrasive when it moves in a deep groove, leading to a higher rate of frictional force. Because the atoms from the protrusion of the machined groove also contribute to the resistance of the workpiece during machining. In addition, the shear stress rises when increasing the machining time. The high-stress zone intrudes deeper and broader when increasing the machining time. The reason is also the accumulation of more atoms covering around the abrasive that require a higher force to be removed.

**Figure 8:**
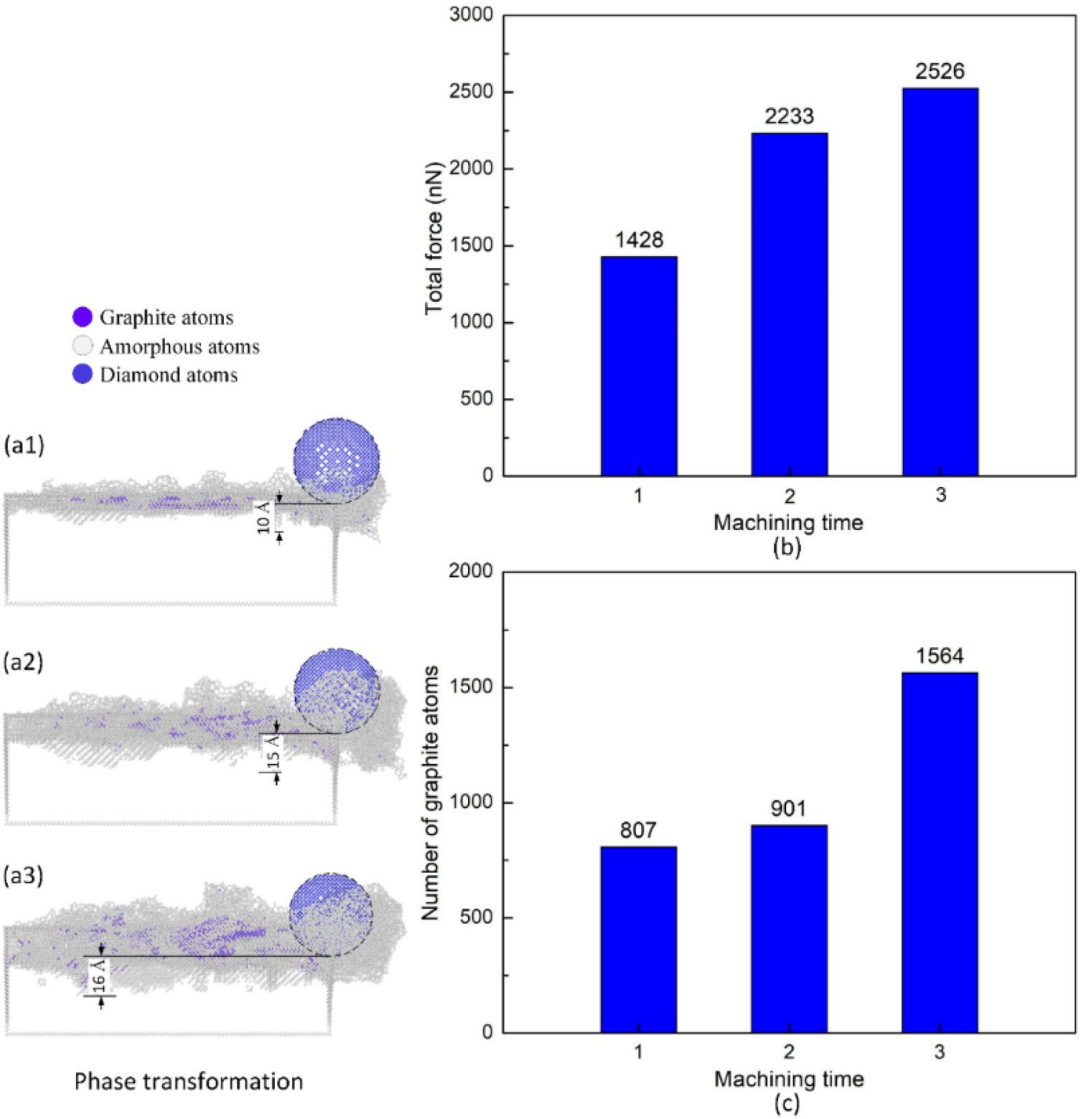
Phase transformation, total force, and number of graphite atoms when applying multi-machining in the vertical direction at 300 K, 100 m/s: (**a1**) first time, (**a2**) second time, and (**a3**) third time; (**b**) total force vs machining time; (**c**) number of graphite atoms vs machining time.

Figure 8a1–a3 show the phase transformation when applying multi-machining in the vertical direction at 300 K and 100 m/s. Initially, the 5 Å depth machining creates a 10 Å SSD depth. When machining for the second and the third times, despite there is an amorphous layer before machining, the abrasive still induces a deeper SSD of 15 Å and 16 Å, respectively. The reason could be the development of a deformation system from the previous machining surface, facilitating the spreading of SSD, as presented gradually from Fig. 8a1–a3. Therefore, different from machining on the perfect flat surface, machining at the same 5 Å depths many times primarily results in a deeper SSD. Figure 8b–c show the total force and the number of graphite atoms when applying multi-machining in the vertical direction at 300 K, 100 m/s. The force values are 1428 nN, 2233 nN, and 2526 nN, corresponding to the first, the second, and the third machining times. The force rises as the machining time increases. After the first machining time, the abrasive would be covered by the round shape of the groove, enhancing the contact area with the substrate material. Therefore, the force for the same 5 Å depth for the following machining times rises gradually. Besides, the numbers of graphite atoms are 807, 901, and 1564 atoms, corresponding to the first, the second, and the third machining times. The number of graphite atoms rises when the depth increases. The numbers calculated for the subsequent machining time also includes the graphite atoms generated in the previous time. Therefore, if the old graphite atoms are ignored, the rate of increasing graphite atoms when machining many times is relatively low. Notably, the increasing rate of graphite atoms in these cases is vastly lower than when raising the polishing depth at the single machining cases, as presented in Fig. 3d. The reason is the difference in the substrate temperature, as shown in Figs. 3a1–a3 and 7b1–b3. These figures reveal that machining many times does not cause a drastic rise in the substrate temperature like single machining on the flat surface. The higher temperature level facilitates the graphitization process, leading to a higher number of graphite atoms in the single machining cases.

Besides examining the multi-machining in the vertical direction, this section also explores this process in the horizontal direction. First, the abrasive moves in the middle of the substrate at 10 Å depth. Then, the abrasive moves in the right direction for 14 Å or 28 Å, as shown in Fig. 1c. Supplementary Fig. 2a–c show the surface morphology when applying multi-machining in the horizontal direction at 300 K and 100 m/s. Moving out of the middle of the first machining groove can cause an accumulation in the protrusion height if the moving distance is short enough, as shown in Supplementary Fig. 2b. While the depths of the grooves of different machining times are nearly the same, fluctuating around 7–8 Å. Interestingly, machining at position C produces some high protrusions or “islets” in the middle of two grooves system due to the further distance from the initial first time A case comparing to the second time B case, as present in Supplementary Fig. 2c.

Supplementary Fig. 3a–c demonstrate the temperature and shear stress distributions when applying multi-machining horizontally at 300 K, 100 m/s. The levels of enhancing temperature and stress and their distribution depend strongly on the polishing position. At the first machining time, the deformation rate is highest with the highest level of pile-up, temperature, and stress distribution. The high-temperature zone also covers around the abrasive regularly. At the second time at position B, the groove presence beside the pathway induces the lowest deformation rate. The temperature distribution at this machining position around the abrasive is not distributed evenly. Because further away from the groove than position B, machining at position C suffers a weaker effect from the initial groove. The pile-up, temperature, and stress levels in position C reduce at a lower rate than position B.

Supplementary Fig. 4 shows the phase transformation, force diagram, and the number of graphite atoms when applying multi-machining in the horizontal direction at 300 K, 100 m/s. Machining in different positions in multi-machining does not generate different SSD depths with a value of 14 Å. Nevertheless, they lead to differences in the force and number of graphite atoms. The total force values are 2254 nN, 2313 nN, and 3253 nN, corresponding to the first time A, the second time B, and the second time C. While the numbers of graphite atoms are 1663, 1667, and 2017 atoms corresponding to the first time A, the second time B, and the second time C, respectively. Position C exhibits a higher rate of deformation than the other positions, as shown in Supplementary Fig. 3a–c. Therefore, the force and the number of graphite atoms are higher than position B.

### Polycrystalline

Besides investigating the single-crystal diamond substrate in the prior sections, this part of the report examines the polycrystalline structure of the diamond substrate to clarify the effect of the grain size on the machining process. The diamond grain size in this section varies in a range of 2–7 nm. The tooltip in this section moves at a speed of 100 m/s, a 10 Å depth, and the workpiece temperature is 300 K.

**Figure 9:**
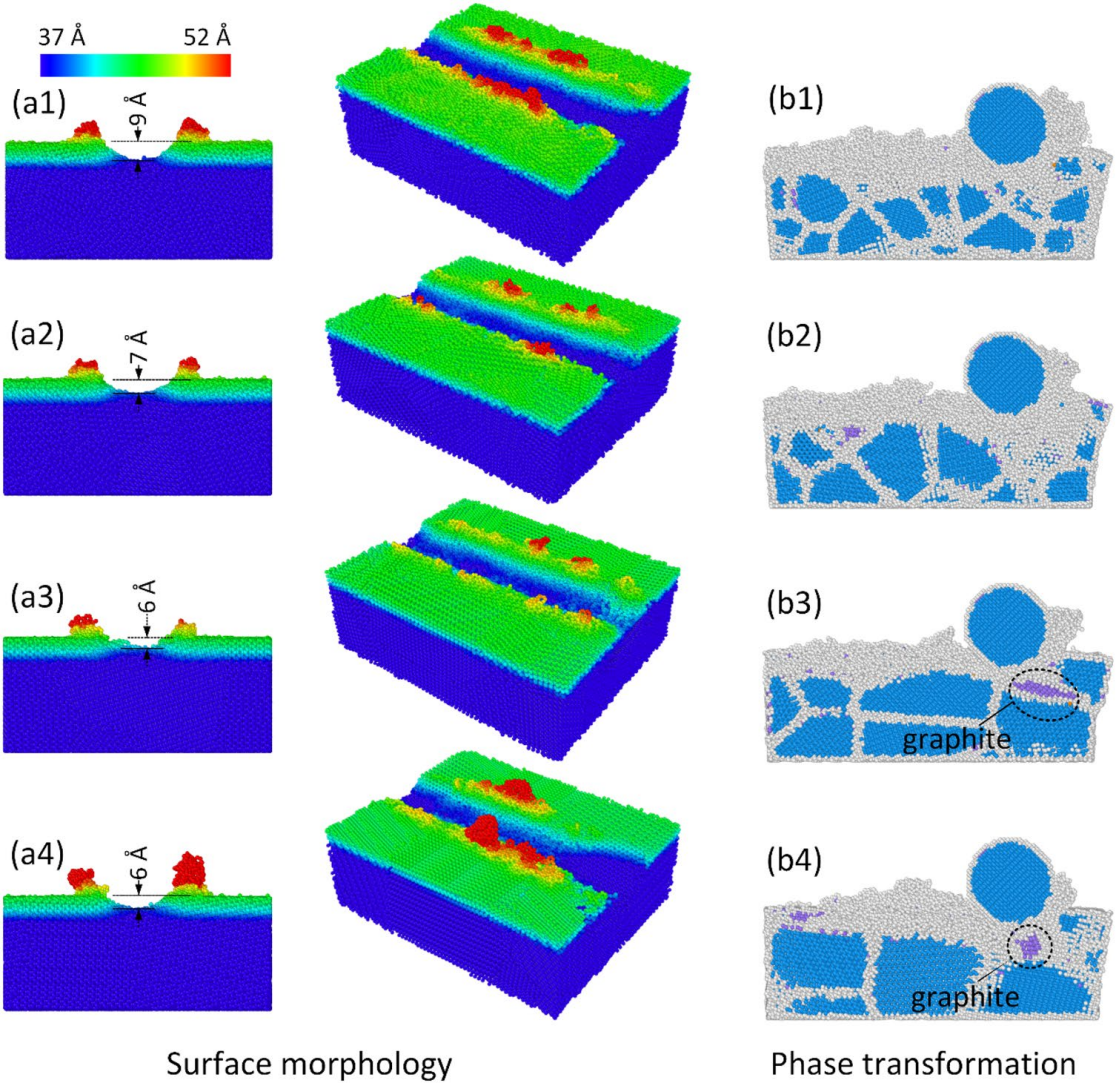
Surface morphology, phase transformation at different grain sizes at 300 K, 100 m/s, and 10 Å depth: (**a1**, **b1**) 2 nm, (**a2**, **b2**) 3 nm, (**a3**, **b3**) 5 nm, and (**a4**, **b4**) 7 nm.

Figure 9a1–a4 show the surface morphology at different grain sizes at 300 K, 100 m/s, and 10 Å depth. The groove depths are 9 Å, 7 Å, 6 Å, and 6 Å corresponding to grain sizes 2 nm, 3 nm, 5 nm, and 7 nm. The larger grain size mostly has the shallower groove depth. In other words, the larger grain size demonstrates a higher elastic capacity because it has a higher grain-boundary ratio, a good result compared to other studies^10,24^. Because the atoms in the grain boundaries exist in a disordered structure, having a lower density than the atoms inside the grain. They can store a lower level of elastic energy comparing to the atoms inside the grain. Increasing the grain size leads to a reduction in the grain boundaries ratio and a rise in the ratio of the crystalline atoms, resulting in a higher level of elastic recovery. This result is similar to the reverse Hall–Petch effect that appeared in nanocrystalline materials that the grain size is smaller than 10 nm^24,25^. In this range, the smaller nanocrystalline diamond grain tends to be easier destroyed comparing to the larger one. Reducing the grain size mostly leads to an increase in the protrusion height, as shown in Fig. 9a1–a4. Because decreasing the nanocrystalline grain size in this range makes the material softer due to the appearance of the grain boundaries. Similar to the single-crystalline diamond substrate, the diamond grains in the polycrystalline structure also experience the graphitization process. Figure 9b1–b4 represent the phase transformation at different grain sizes at 300 K, 100 m/s, and 10 Å depth. Remarkably, the larger grain size can create a bigger graphite cluster. The graphitization process need a diamond structure to happen, therefore, a larger diamond grain size can form a larger graphite cluster.

**Figure 10:**
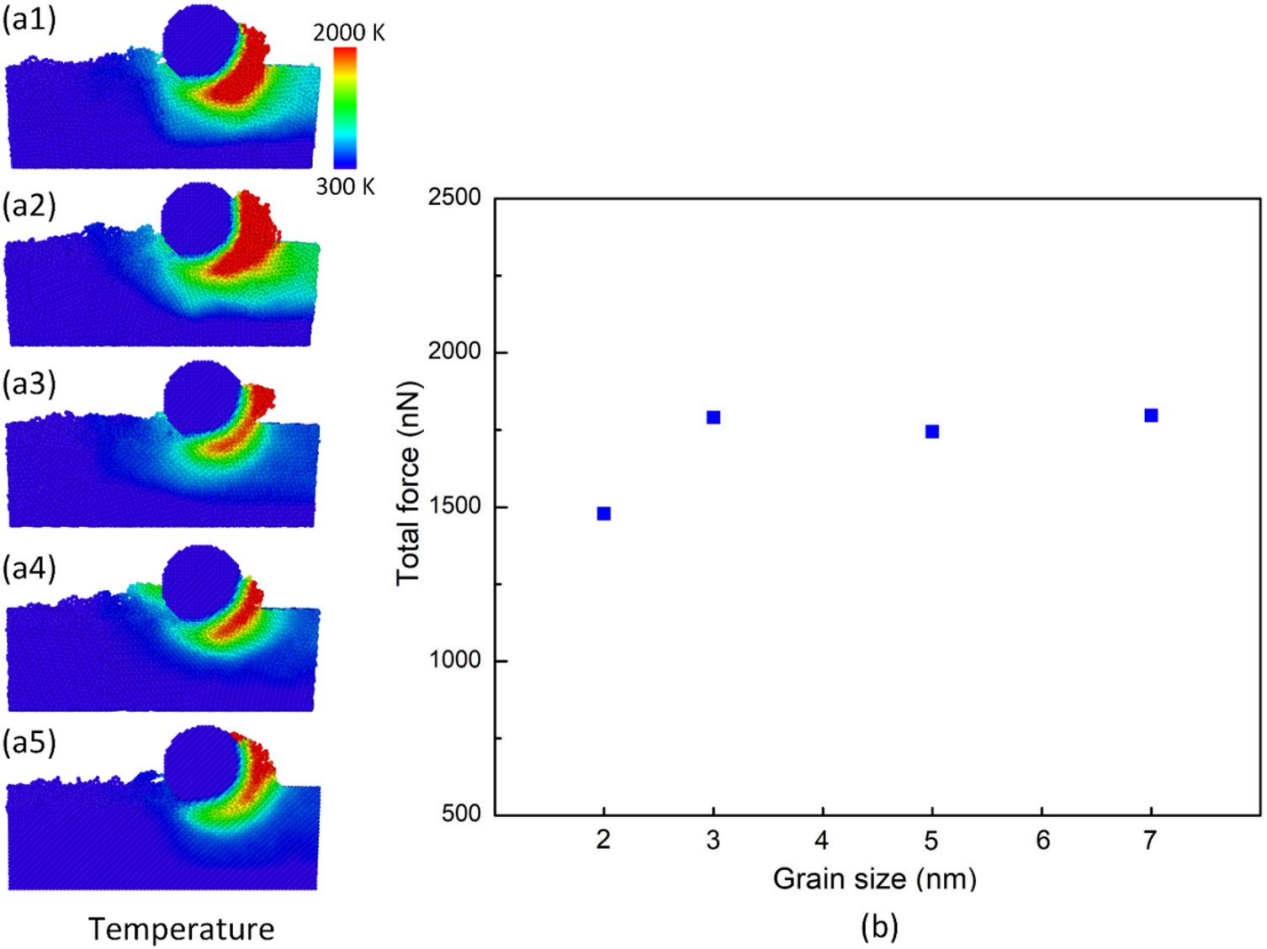
Temperature distribution and total force at different grain sizes at 300 K, 100 m/s, and 10 Å depth: (**a1**) 2 nm, (**a2**) 3 nm, (**a3**) 5 nm, and (**a4**) 7 nm; (**b**) the total force vs grain size.

Figure 10a1–a4 exhibit the temperature distribution at different grain sizes at 300 K, 100 m/s, and 10 Å depth. Generally, the smaller grain size creates a strongly higher substrate temperature. The 2 nm and 3 nm grain sizes exhibit a dramatically high-temperature zone, as shown in Fig. 10a1,a2. While the 5 nm and 7 nm grain sizes generate a similar temperature increase, which is much lower than the 2 nm and 3 nm grain sizes. The existence of more grain boundaries in the smaller grain size hinders the continuity of the force transformation. In the small grain size, the grain boundary sliding dominates the deformation mechanism rather than the dislocation^26^. Moreover, with the nanocrystalline materials that the grain size is smaller than 10–15 nm, reducing the grain size leads to a decline in the mechanical properties, following the inverse Hall–Petch relation^27^. Therefore, the atoms in the smaller grain size are stronger moved and mixed, leading to a higher temperature. Figure 10b present the total force at different grain sizes at 300 K, 100 m/s, and 10 Å depth. The total forces are 1479 nN, 1789 nN, 1744 nN, and 1796 nN, corresponding to grain sizes 2 nm, 3 nm, 5 nm, and 7 nm. The force values of 3–7 nm grains are similar. When the grain size declines to 2 nm, the force drops rapidly. It means that the 2 nm grain size requires the lowest force. The smaller grain size mostly needs a smaller force to be deformed. In the smaller grain size, the crystalline structure is divided into smaller zones. The reduction in the nanocrystalline grain size will soften the substrate material due to the surge of the grain-boundary ratio, a consistent result to Remediakis et al.^28^ and Sha et al.^29^ studies.

We survey the effect of the speed on the machining process. Supplementary Fig. 5 shows the shear strain and shear stress distributions at different speeds at 300 K and 10 Å depth. Overall, the higher machining speed can generate a more extended protrusion along two sides of the pathway of the tooltip, as shown in Supplementary Fig. 5a1–d1. Because at an ultra-high-speed, the materials tend to become more ductile, leading to a higher rate of deformation. On the other hand, the shear stress is not sensitive to the machining velocity, as presented in Supplementary Fig. 5a2–d2, a good result compared to Varga et al. report^30^. Moreover, the pile-up shape in front of the tooltip becomes more disordered when increasing the machining speed. The reason is when increasing the machining speed, the more significant amount of the kinetic energy that the pile-up absorbs from the tooltip movement.

Supplementary Fig. 6 illustrates the temperature distribution and phase transformation of different speeds at 300 K, 10 Å depth. Although the shear stress does not strongly depend on the machining speed, the temperature of the diamond substrate is certainly sensitive to it. The rise in the machining speed induces a significantly higher substrate temperature, as represented in Supplementary Fig. 6a1–d1. The reason is the kinetic energy of the tooltip is intensely transferred to the substrate atoms, causing a significant rise in its temperature. Supplementary Fig. 6a2–d2 indicate that the graphite atoms intermixed with the amorphous atoms. The graphitization appears when the stress applied by the tooltip is high enough, while the high-temperature appearance makes this process easier^16,20^. The SSD thickness oscillates around 14–15 Å. The SSD value and pile-up height do not have an appreciable change when improving the machining speed.

Supplementary Fig. 7a,b illustrate the total force and number graphite atoms at different speeds at 300 K, 10 Å depth. The total force values are 2475 nN, 2254 nN, 2195 nN, and 2072 atoms corresponding to 50 m/s, 100 m/s, 200 m/s, and 400 m/s, respectively. The higher speed generates the lower force due to the higher substrate temperature at a higher speed^31,32^. Due to the increasing bonding length when raising the temperature leading to the lower bonding level, the higher substrate temperature requires a lower force to machining. The numbers of graphite atoms are 2021, 1663, 1278, and 1606 atoms corresponding to 50 m/s, 100 m/s, 200 m/s, and 400 m/s, respectively. From 50 to 200 m/s, the number of graphene atoms declines when raising the speed due to the reduction in the force. However, at 400 m/s, when the tip induces an extremely higher temperature in the substrate, as shown in Supplementary Fig. 6d1, the number of graphite atoms rises. The higher temperature will trigger the higher rate of phase transformation from diamond to graphite structure^33^. In this case, the effect of temperature improvement excesses the effect of force reduction. Thus, the number of graphite atoms depends on the machining speed and the improvement of the substrate temperature.

## Discussion

In summary, this report reveals the effects of machining depth, velocity, temperature, multi-machining, and grain size on the deformation behaviours and graphitization process of a diamond substrate. The results show that improving the machining depth results in a higher deformation rate, stress, substrate temperature, and the number of graphite atoms. Furthermore, at 15 Å depth, the appearance of graphite sheets can lead to a sudden drop in the total force. Increasing the diamond substrate temperature results in decline in the force as the softening of the material. Besides, the number of graphite atoms grows dramatically as the temperature rises because the graphitization happens stronger. Machining in the vertical direction for multi-time gives rise to the force, temperature, stress, pile-up, and SSD thickness because more substrate atoms cover around the tooltip. However, the number of graphite atoms when machining many times is considerably lower than in the single machining cases. Because the presence of the amorphous and graphite atoms in the previous machining time reduces the impact of the tooltip on the substrate. Improving the nanocrystalline grain size could induce a larger graphite cluster, higher rates of elastic recovery, and temperature but a lower force and pile-up height. Because reducing the grain size will increase the grain-boundary ratio and soften the substrate material, following the inverse Hall–Petch relation. Increasing the machining time in the horizontal direction leads to the accumulation of protrusion. While the temperature, stress, and number of graphite atoms rise when the tooltip moves further from the first machining position. The stress, pile-up, SSD values are not strongly affected by the machining speed. Otherwise, the higher machining speed produces a higher substrate temperature and a lower force as the substrate is softened due to the temperature improvement. To minimize the damage formation, reducing the machining depth is the key factor. Besides, machining in orientation (100)[110] could also limit subsurface damage formation comparing to other orientations.

## Method

The simulation model consists of a diamond workpiece and a tooltip, as shown in Fig. 1a–c. The tooltip machines in (001) face, along [100] direction of the diamond workpiece. The details of the simulation parameters are listed in Table 1. The depth is examined in a range of 5–15 Å to characterize the different levels of machining. During the machining process, the frictional force can cause a dramatic increase in the substrate temperature^18,34,35^. Therefore, this report surveys the effect of substrate temperature from 300 to 1200 K on the machining process. Ordinarily, most studies decide to choose a perfectly flat surface^36–38^, a rough surface^39–41^, or a surface covered by a thin amorphous/oxidation layer^42–44^ to investigate the tribological properties. This report tries to examine both a perfect flat surface and a deformed surface by machining for one time or multi-times, as shown in Fig. 1b. Finally, the structure of a diamond film can exist in both single and polycrystalline^45,46^; therefore, the report investigates both these two types of crystal structure, as shown in Fig. 1a–c.

**Table 1:** Parameters of simulation in the machining process.

Parameters	Details
Dimensions workpiece (Å)	150 × 50 × 120
Tooltip	Diamond, diameter 40 Å
Depth (Å)	5, 10, 15
Substrate temperature (K)	300, 600, 900, 1200
Machining velocity (m/s)	50, 100, 200, 400
Machining time	1 time, 3 times
Crystal	Single, polycrystalline
Orientation	(100)[100], (100)[110], (110)[100], (110)[110]
Time step	1.0 femtosecond

To describe the bonding between C–C atoms, a popular potential function called Tersoff (an analytical bond-order potential or ABOP version) that can simulate the phase transformation from diamond to amorphous and graphite structures is implied in this study^9,10,47–49^. We select an open-source software named LAMMPS and OVITO to simulate and explore the model^50,51^.

## Supplementary Information

Supplementary Figures.

## Data Availability

The data in this manuscript is available upon reasonable request.

## References

[1] Alcantar-Peña JJ (2019). Polycrystalline diamond films with tailored micro/nanostructure/doping for new large area film-based diamond electronics. Diam. Relat. Mater..

[2] Auciello O, Sumant AV (2010). Status review of the science and technology of ultrananocrystalline diamond (UNCD^TM^) films and application to multifunctional devices. Diam. Relat. Mater..

[3] Sumant AV (2005). Ultrananocrystalline diamond film as a wear-resistant and protective coating for mechanical seal applications. Tribol. Trans..

[4] Liu WL (2006). Thermal conduction in nanocrystalline diamond films: Effects of the grain boundary scattering and nitrogen doping. Appl. Phys. Lett..

[5] Umezawa H, Mokuno Y, Yamada H, Chayahara A, Shikata S-I (2010). Characterization of Schottky barrier diodes on a 0.5-inch single-crystalline CVD diamond wafer. Diamond Relat. Mater..

[6] Ichikawa K, Kurone K, Kodama H, Suzuki K, Sawabe A (2019). High crystalline quality heteroepitaxial diamond using grid-patterned nucleation and growth on Ir. Diam. Relat. Mater..

[7] Irifune T, Kurio A, Sakamoto S, Inoue T, Sumiya H (2003). Ultrahard polycrystalline diamond from graphite. Nature.

[8] Yuan S (2019). Sub-nanoscale polishing of single crystal diamond(100) and the chemical behavior of nanoparticles during the polishing process. Diamond Relat. Mater..

[9] Zong W, Cheng X, Zhang J (2016). Atomistic origins of material removal rate anisotropy in mechanical polishing of diamond crystal. Carbon.

[10] He G, Xu C, Liu C, Liu H, Wang H (2019). Grain size and temperature effects on the indentation induced plastic deformations of nano polycrystalline diamond. Appl. Surf. Sci..

[11] Thomas ELH, Nelson GW, Mandal S, Foord JS, Williams OA (2014). Chemical mechanical polishing of thin film diamond. Carbon.

[12] Lu J, Xiao P, Tong R, Luo Q, Xu X (2019). Precision polishing of single crystal diamond (111) substrates using a Sol-gel (SG) polishing Pad. IEEE Trans. Semiconductor Manufact..

[13] Yang N, Zong WJ, Li ZQ, Sun T (2014). Amorphization anisotropy and the internal of amorphous layer in diamond nanoscale friction. Comput. Mater. Sci..

[14] Roy S (2018). A comprehensive study of mechanical and chemo-mechanical polishing of CVD diamond. Mater. Today Proc..

[15] Yang N, Huang W, Lei D (2018). The effects of diamond amorphous layer on the diamond lapping surface. Procedia CIRP.

[16] Gogotsi YG, Kailer A, Nickel KG (1999). Transformation of diamond to graphite. Nature.

[17] Pham A-V, Fang T-H, Nguyen V-T, Chen T-H (2021). Effect of incidence and size of graphite particle on the formation of graphene on Ni surfaces. Vacuum.

[18] Pham V-T, Fang T-H (2020). Pile-up and heat effect on the mechanical response of SiGe on Si(0 0 1) substrate during nanoscratching and nanoindentation using molecular dynamics. Comput. Mater. Sci..

[19] Ren G, Zhang D, Gong X (2011). Dynamical multiscale simulation of nanoindentation. Phys. Lett. A.

[20] Fan P (2021). Molecular dynamics simulation of AFM tip-based hot scratching of nanocrystalline GaAs. Mater. Sci. Semiconductor Process..

[21] Chien C-H (2016). Temperature effect on kinetic friction characteristics of Cu substrate composed by single crystal and polycrystalline structures. Comput. Mater. Sci..

[22] Pham V-T, Fang T-H (2021). Influences of grain size, alloy composition, and temperature on mechanical characteristics of Si_100__−__x_Gex alloys during indentation process. Mater. Sci. Semiconductor Process..

[23] Qiu C, Zhu P, Fang F, Yuan D, Shen X (2014). Study of nanoindentation behavior of amorphous alloy using molecular dynamics. Appl. Surf. Sci..

[24] Li J (2020). Nanoindentation response of nanocrystalline copper via molecular dynamics: Grain-size effect. Mater. Chem. Phys..

[25] Doan D-Q, Fang T-H, Chen T-H (2020). Influences of grain size and temperature on tribological characteristics of CuAlNi alloys under nanoindentation and nanoscratch. Int. J. Mech. Sci..

[26] Ma Z, Gamage RP, Zhang C (2021). Effects of temperature and grain size on the mechanical properties of polycrystalline quartz. Comput. Mater. Sci..

[27] Naik SN, Walley SM (2019). The Hall-Petch and inverse Hall-Petch relations and the hardness of nanocrystalline metals. J. Mater. Sci..

[28] Remediakis IN, Kopidakis G, Kelires PC (2008). Softening of ultra-nanocrystalline diamond at low grain sizes. Acta Mater..

[29] Sha Z, Branicio P, Sorkin V, Pei Q, Zhang Y (2011). Effects of grain size and temperature on mechanical and failure properties of ultrananocrystalline diamond. Diam. Relat. Mater..

[30] Varga M, Leroch S, Eder SJ, Rojacz H, Ripoll MR (2019). Influence of velocity on high-temperature fundamental abrasive contact: A numerical and experimental approach. Wear.

[31] Thakur DG, Ramamoorthy B, Vijayaraghavan L (2009). Study on the machinability characteristics of superalloy Inconel 718 during high speed turning. Mater. Des..

[32] Pawade RS, Joshi SS, Brahmankar PK, Rahman M (2007). An investigation of cutting forces and surface damage in high-speed turning of Inconel 718. J. Mater. Process. Technol..

[33] Xiang J, Xie L, Gao F, Yi J, Pang S, Wang X (2018). Diamond tools wear in drilling of SiCp/Al matrix composites containing Copper. Ceram. Int..

[34] Li B, Clapp P, Rifkin J, Zhang X (2003). Molecular dynamics calculation of heat dissipation during sliding friction. Int. J. Heat Mass Transf..

[35] Tong R-T, Han B, Quan Z-F, Liu G (2019). Molecular dynamics simulation of friction and heat properties of Nano-texture GOLD film in space environment. Surf. Coat. Technol..

[36] Si L, Guo D, Luo J, Lu X (2010). Monoatomic layer removal mechanism in chemical mechanical polishing process: A molecular dynamics study. J. Appl. Phys..

[37] Goel S, Luo X, Reuben RL (2013). Wear mechanism of diamond tools against single crystal silicon in single point diamond turning process. Tribol. Int..

[38] Han X, Hu Y, Yu S (2009). Investigation of material removal mechanism of silicon wafer in the chemical mechanical polishing process using molecular dynamics simulation method. Appl. Phys. A.

[39] Agrawal PM, Raff LM, Bukkapatnam S, Komanduri R (2010). Molecular dynamics investigations on polishing of a silicon wafer with a diamond abrasive. Appl. Phys. A.

[40] Nguyen V-T, Fang T-H (2020). Molecular dynamics simulation of abrasive characteristics and interfaces in chemical mechanical polishing. Appl. Surface Sci..

[41] Nguyen V-T, Fang T-H (2020). Material removal and interactions between an abrasive and a SiC substrate: A molecular dynamics simulation study. Ceram. Int..

[42] Chen J (2018). Effect of indentation speed on deformation behaviors of surface modified silicon: A molecular dynamics study. Comput. Mater. Sci..

[43] Nguyen V-T, Fang T-H (2020). Abrasive mechanisms and interfacial mechanics of amorphous silicon carbide thin films in chemical-mechanical planarization. J. Alloys Compounds.

[44] Nguyen V-T, Fang T-H (2020). Material removal and wear mechanism in abrasive polishing of SiO_2_/SiC using molecular dynamics. Ceram. Int..

[45] Shikata S (2016). Single crystal diamond wafers for high power electronics. Diam. Relat. Mater..

[46] Benabdesselam M, Iacconi P, Butler J, Nigoul J (2003). TL characterisation of a CVD diamond wafer for ionising radiation dosimetry. Diam. Relat. Mater..

[47] Dong G, Wang X, Gao S (2018). Molecular dynamics simulation and experiment research of cutting-tool wear mechanism for cutting aluminum alloy. Int. J. Adv. Manuf. Technol..

[48] Zhao J, Zhang C, Liu F, Cheng GJ (2021). Understanding femtosecond laser internal scribing of diamond by atomic simulation: Phase transition, structure and property. Carbon.

[49] Narulkar R, Bukkapatnam S, Raff L, Komanduri R (2009). Graphitization as a precursor to wear of diamond in machining pure iron: A molecular dynamics investigation. Comput. Mater. Sci..

[50] Plimpton S (1995). Fast parallel algorithms for short-range molecular dynamics. J. Comput. Phys..

[51] Stukowski A (2009). Visualization and analysis of atomistic simulation data with OVITO—The Open Visualization Tool. Model. Simul. Mater. Sci. Eng..

