# Supplementary material for: Phase transformation and subsurface damage formation in the ultrafine machining process of a diamond substrate through atomistic simulation
Source: Sci Rep. 2021 Sep 7;11:17795. doi: 10.1038/s41598-021-97419-9 (PMC8423824; doi:10.1038/s41598-021-97419-9)
Supplement: Supplementary file 1 — Supplementary Figures. [file 41598_2021_97419_MOESM1_ESM.pdf]

Phase transformation and subsurface damage formation in the ultrafine machining process of a diamond substrate through atomistic simulation

Van-Thuc Nguyen[1,2], Te-Hua Fang[1,*]

[1]Department of Mechanical Engineering, National Kaohsiung University of Science and Technology, Kaohsiung 807, Taiwan

[2]Faculty of Mechanical Engineering, Ho Chi Minh City University of Technology and Education, Ho Chi Minh City, Vietnam

[*]Corresponding author, email: fang.tehua@msa.hinet.net.

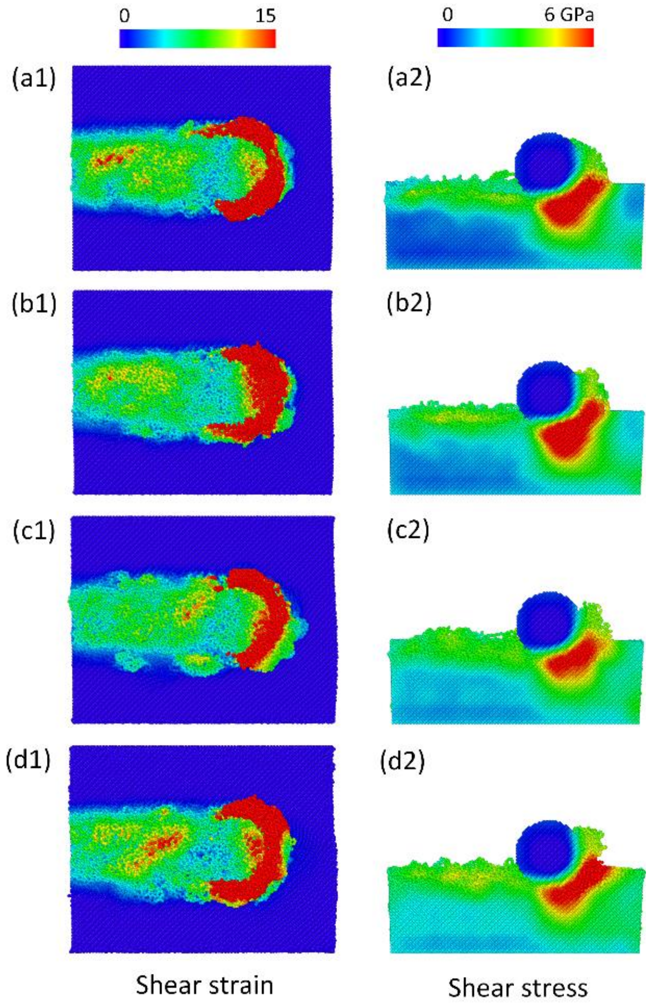

Supplementary Fig. 1. Shear strain and shear stress distributions of different temperatures at 100 m/s and 10 Å depth: (a1)-(a2) 300 K, (b1)-(b2) 600 K, (c1)-(c2) 900 K, and (d1)-(d2) 1200 K.

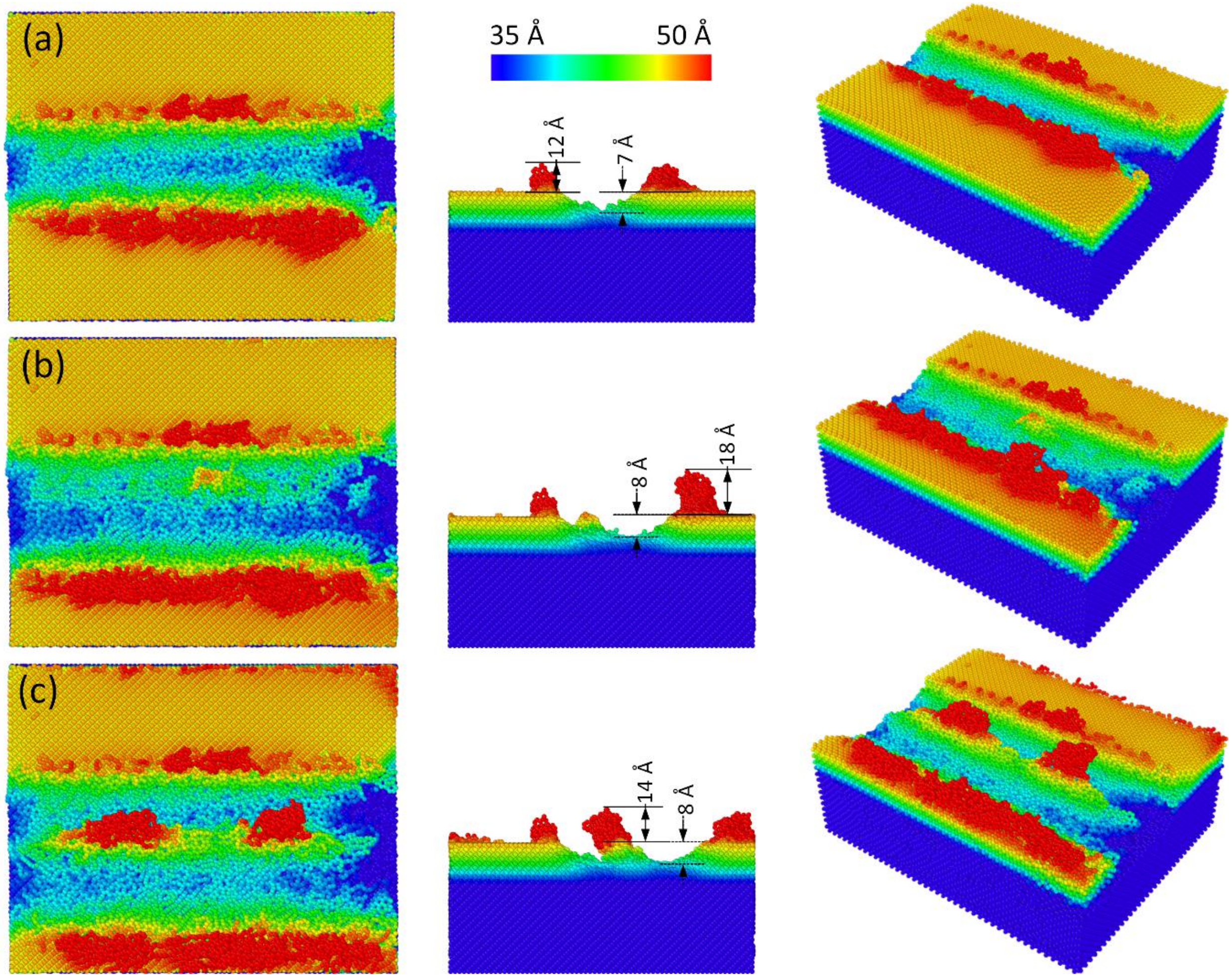

Supplementary Fig. 2. Surface morphology when applying multi-machining in horizontal direction at 300 K and 100 m/s: (a) first time A, (b) second time B, and (c) second time C.

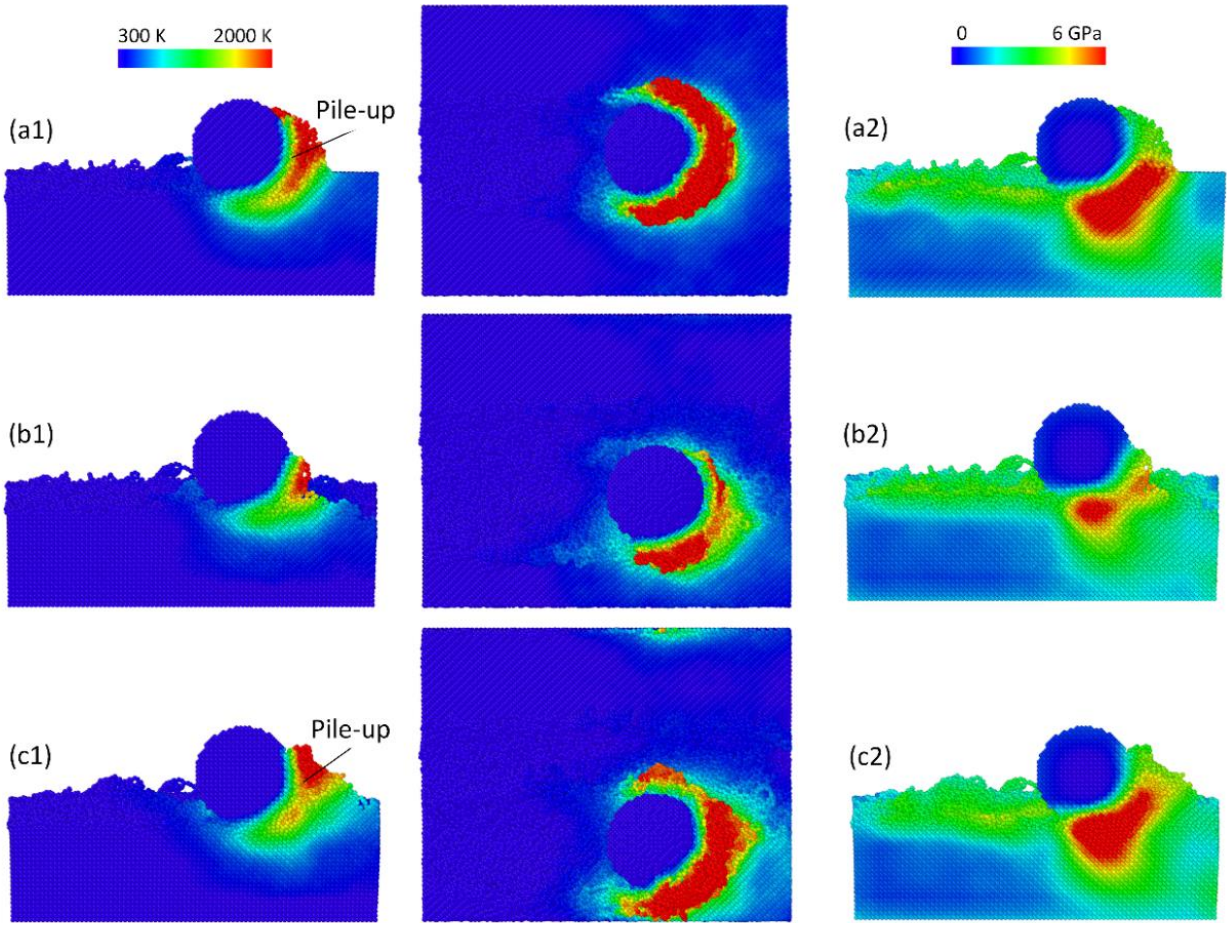

Supplementary Fig. 3 Temperature and shear stress distributions when applying multi-machining in horizontal direction at 300 K, 100 m/s: (a) first time A, (b) second time B, and (c) second time C.

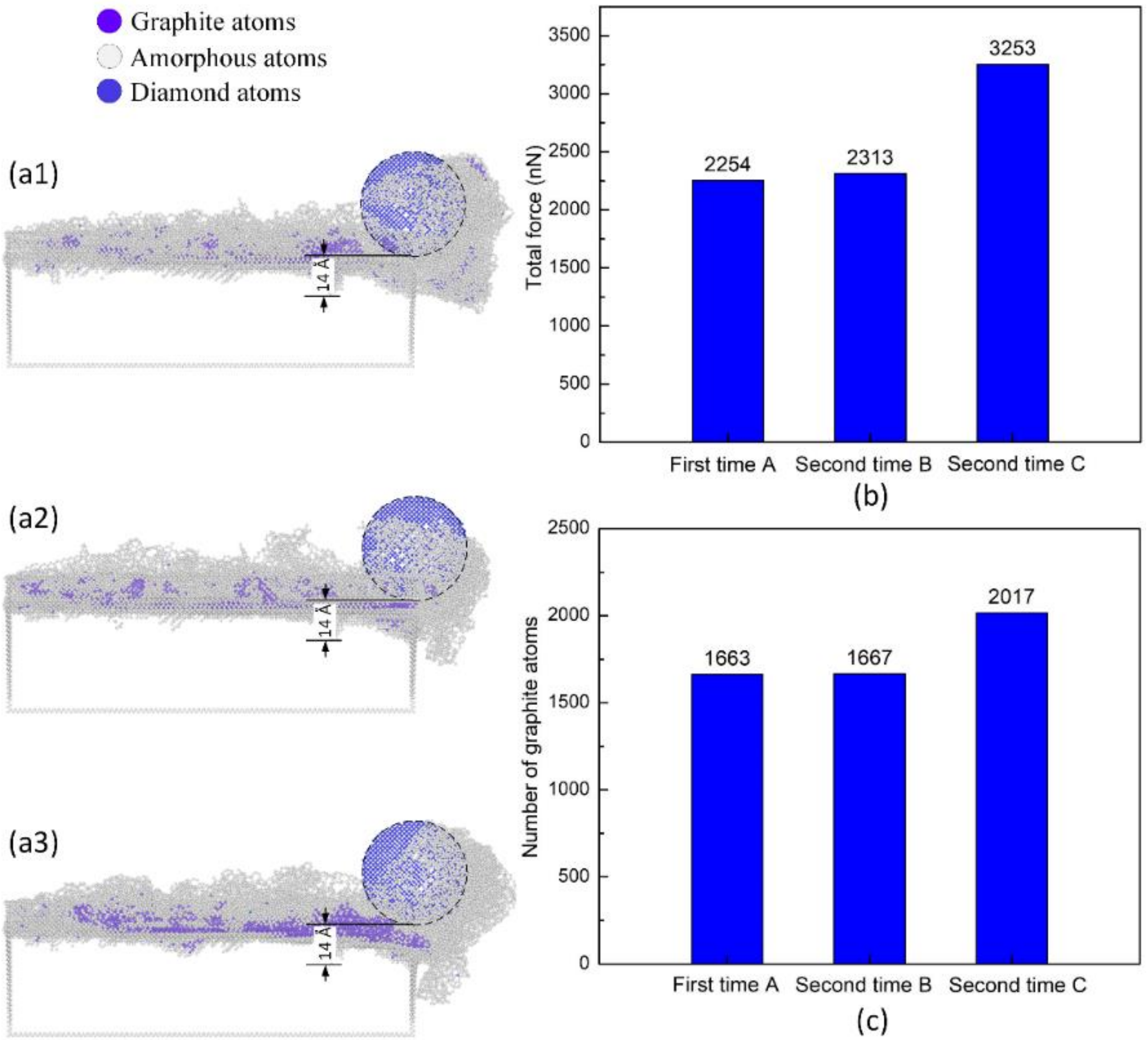

Supplementary Fig. 4. Phase transformation, force diagram, and number of graphite atoms when applying multi-machining in horizontal direction at 300 K, 100 m/s: (a1) first time A, (à) second time B, and (a3) second time C; (b) force diagram vs machining time; (c) number graphite atoms vs machining time.

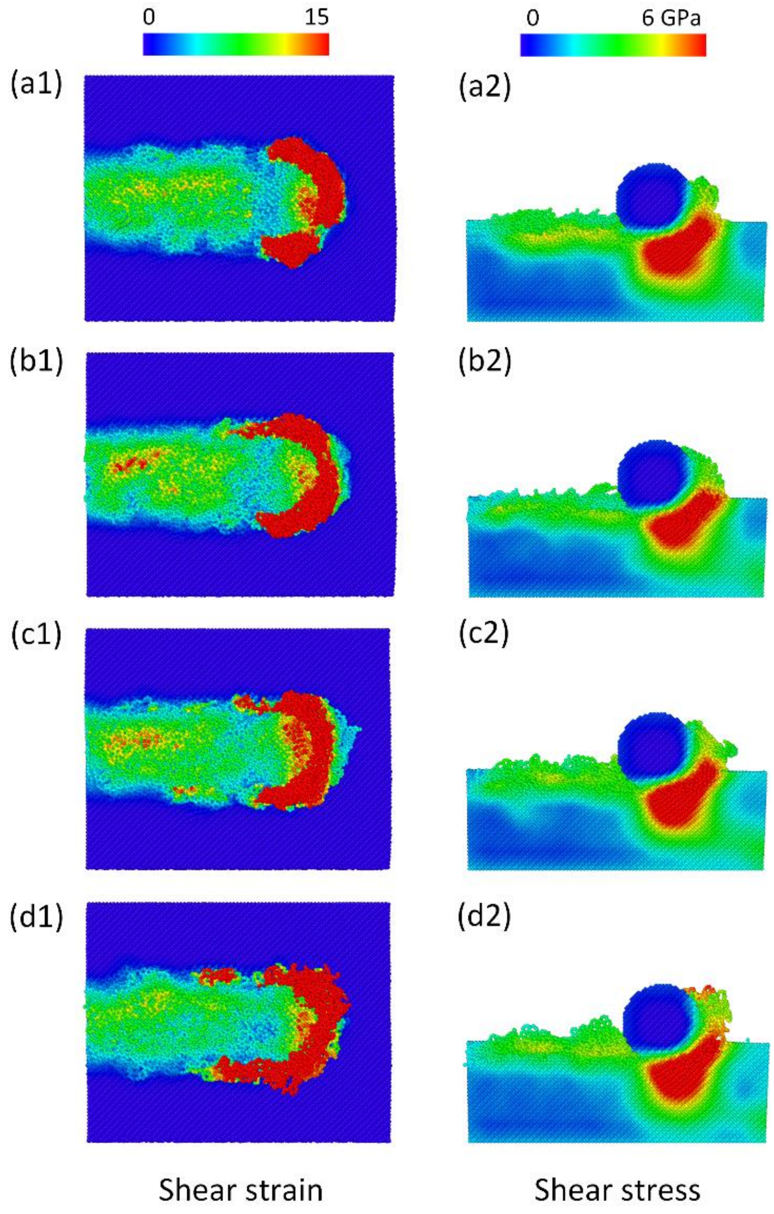

Supplementary Fig. 5. Shear strain and shear stress distributions of different speeds at 300 K and 10 Å depth: (a1)-(a2) 50 m/s, (b1)-(b2) 100 m/s, (c1)-(c2) 200 m/s, and (d1)-(d2) 400 m/s.

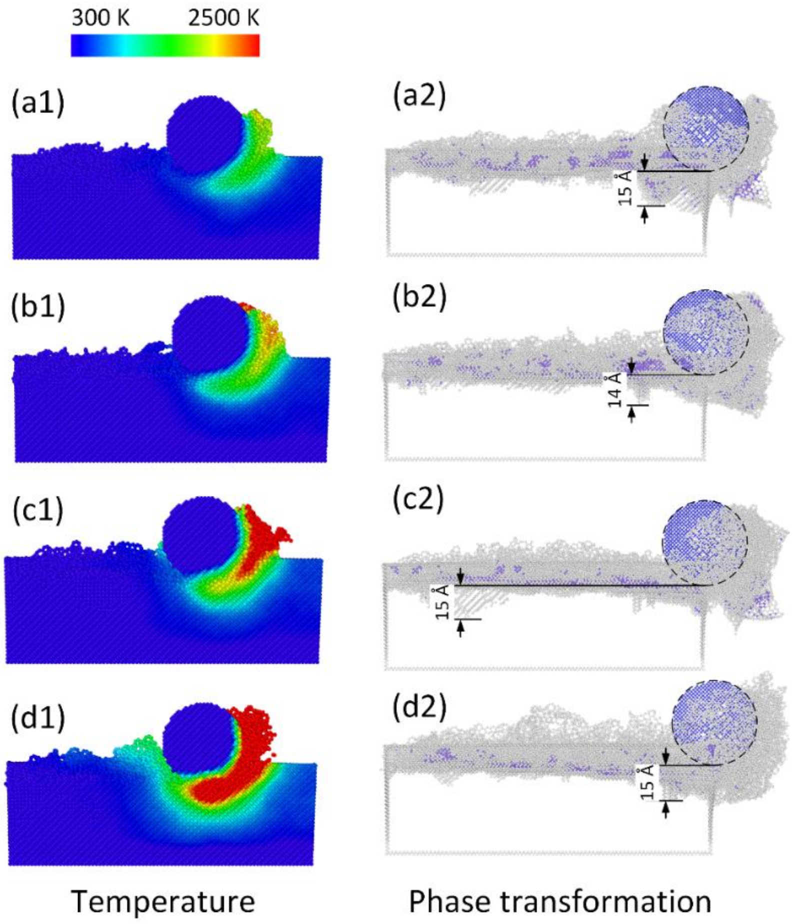

Supplementary Fig. 6. Temperature distribution and phase transformation of different speeds at 300 K, 10 Å depth: (a1)-(a2) 50 m/s, (b1)-(b2) 100 m/s, (c1)-(c2) 200 m/s, and (d1)-(d2) 400 m/s.

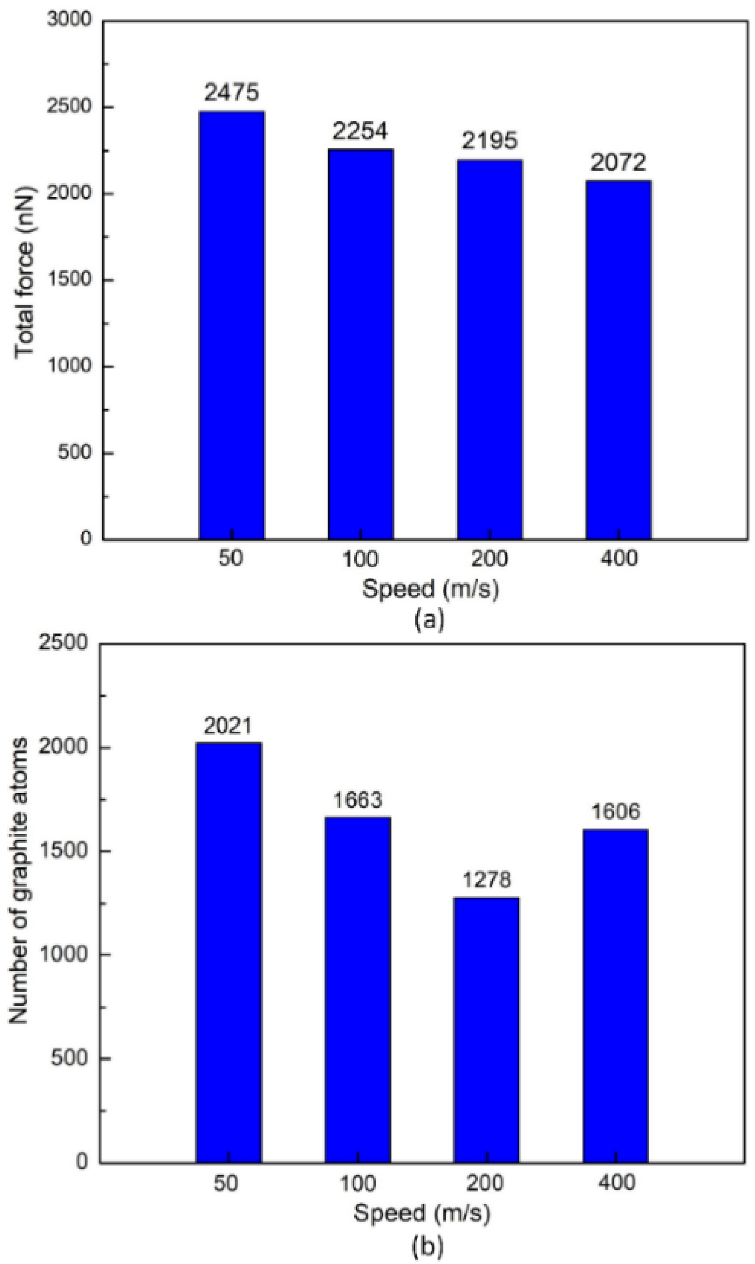

Supplementary Fig. 7. Total force and number of graphite atoms at different speeds at 300 K, 10 Å depth: (a) total force, and (b) number of graphene atoms.
